# Supplementary material for: Robust bacterial co-occurence community structures are independent of r- and K-selection history
Source: Sci Rep. 2021 Dec 6;11:23497. doi: 10.1038/s41598-021-03018-z (PMC8648916; doi:10.1038/s41598-021-03018-z)
Supplement: Supplementary file 1 — Supplementary Information. [file 41598_2021_3018_MOESM1_ESM.pdf]

Supplementary material for ”Robust bacterial co-occurence community structures are independent of r- and K-selection history”

Jakob Peder Pettersen, Madeleine Gundersen, Eivind Almaas

Contents

S1	Results from main parameter combinations	2
S2	Robustness to random noise	5
S3	Robustness to change of abundance type	10
S4	Robustness to OTU filtering cutoff	15
S5	Effect of similarity measure	20

S1 Results from main parameter combinations

Table S1 shows the OTUs present in modules 3 and 4 in the community labelled network (Fig. 2) in the main article. Figure S1 presents dynamic visualizations of the overall network for low nutrient supply.

Table S1. The OTUs found in module 3 and 4 of Fig. 2 together with their taxonomies and overall mean relative abundances.

Module	Mean abundance	Taxonomy
3	0.1936535	d_Bacteria,p_Proteobacteria,c_Alphaproteobacteria,o_Rhodobacterales,f_Rhodobacteraceae,g_Roseovarius
3	0.0511976	d_Bacteria,p_Bacteroidetes,c_Flavobacteriia,o_Flavobacteriales,f_Flavobacteriaceae,g_Polaribacter
3	0.0398306	d_Bacteria,p_Proteobacteria,c_Alphaproteobacteria,o_Rhodobacterales,f_Rhodobacteraceae,g_Sulfitobacter
3	0.0314970	d_Bacteria,p_Proteobacteria,c_Alphaproteobacteria,o_Rhodobacterales,f_Rhodobacteraceae,uncl_f_Rhodobacteraceae
3	0.0270573	d_Bacteria,p_Firmicutes,c_Bacilli,o_Bacillales,f_Bacillaceae_1,g_Bacillus
3	0.0232589	d_Bacteria,p_Proteobacteria,c_Alphaproteobacteria,o_Rhodobacterales,f_Rhodobacteraceae,uncl_f_Rhodobacteraceae
3	0.0136061	d_Bacteria,p_Proteobacteria,c_Gammaproteobacteria,o_Alteromonadales,f_Alteromonadaceae,g_Marinobacter
3	0.0083859	d_Bacteria,p_Proteobacteria,c_Alphaproteobacteria,o_Rhodobacterales,f_Rhodobacteraceae,g_Lentibacter
3	0.0078133	d_Bacteria,p_Proteobacteria,c_Alphaproteobacteria,o_Rhodobacterales,f_Rhodobacteraceae,uncl_f_Rhodobacteraceae
3	0.0060186	d_Bacteria,p_Proteobacteria,c_Alphaproteobacteria,o_Rhodobacterales,f_Rhodobacteraceae,uncl_f_Rhodobacteraceae
3	0.0034279	d_Bacteria,p_Proteobacteria,c_Alphaproteobacteria,o_Rhodobacterales,f_Rhodobacteraceae,uncl_f_Rhodobacteraceae
3	0.0029334	d_Bacteria,p_Bacteroidetes,c_Flavobacteriia,o_Flavobacteriales,f_Flavobacteriaceae,uncl_f_Flavobacteriaceae
3	0.0026583	d_Bacteria,p_Proteobacteria,c_Alphaproteobacteria,o_Rhodobacterales,f_Rhodobacteraceae,g_Loktanella
3	0.0025617	d_Bacteria,p_Bacteroidetes,c_Flavobacteriia,o_Flavobacteriales,f_Flavobacteriaceae,g_Muricauda
3	0.0021346	d_Bacteria,p_Bacteroidetes,c_Flavobacteriia,o_Flavobacteriales,f_Flavobacteriaceae,g_Aquimarina
3	0.0019096	d_Bacteria,p_Proteobacteria,c_Alphaproteobacteria,o_Rhodobacterales,f_Rhodobacteraceae,uncl_f_Rhodobacteraceae
3	0.0016933	d_Bacteria,p_Proteobacteria,c_Alphaproteobacteria,o_Rhodobacterales,f_Rhodobacteraceae,uncl_f_Rhodobacteraceae
3	0.0015536	d_Bacteria,p_Proteobacteria,c_Alphaproteobacteria,o_Rhodobacterales,f_Rhodobacteraceae,uncl_f_Rhodobacteraceae
3	0.0011400	d_Bacteria,p_Proteobacteria,c_Alphaproteobacteria,o_Rhodobacterales,f_Rhodobacteraceae,uncl_f_Rhodobacteraceae
3	0.0010513	d_Bacteria,p_Proteobacteria,c_Alphaproteobacteria,o_Rhodobacterales,f_Rhodobacteraceae,uncl_f_Rhodobacteraceae
3	0.0010176	d_Bacteria,p_Bacteroidetes,c_Flavobacteriia,o_Flavobacteriales,f_Flavobacteriaceae,g_Marixanthomonas
3	0.0009847	d_Bacteria,p_Proteobacteria,c_Alphaproteobacteria,o_Rhodobacterales,f_Rhodobacteraceae,uncl_f_Rhodobacteraceae
3	0.0009293	d_Bacteria,p_Bacteroidetes,c_Flavobacteriia,o_Flavobacteriales,f_Flavobacteriaceae,uncl_f_Flavobacteriaceae
3	0.0008849	d_Bacteria,p_Proteobacteria,uncl_p_Proteobacteria,uncl_p_Proteobacteria,uncl_p_Proteobacteria,uncl_p_Proteobacteria
3	0.0007680	d_Bacteria,p_Proteobacteria,c_Alphaproteobacteria,o_Rhodobacterales,f_Rhodobacteraceae,uncl_f_Rhodobacteraceae
3	0.0006107	d_Bacteria,p_Bacteroidetes,c_Flavobacteriia,o_Flavobacteriales,f_Flavobacteriaceae,uncl_f_Flavobacteriaceae
3	0.0005083	d_Bacteria,p_Proteobacteria,c_Alphaproteobacteria,o_Rhodobacterales,f_Rhodobacteraceae,g_Sulfitobacter
3	0.0004548	d_Bacteria,p_Proteobacteria,c_Alphaproteobacteria,o_Rhodobacterales,f_Rhodobacteraceae,uncl_f_Rhodobacteraceae
3	0.0004219	d_Bacteria,p_Proteobacteria,c_Alphaproteobacteria,o_Rhodobacterales,f_Rhodobacteraceae,uncl_f_Rhodobacteraceae
3	0.0003926	d_Bacteria,p_Proteobacteria,c_Alphaproteobacteria,o_Rhodobacterales,f_Rhodobacteraceae,uncl_f_Rhodobacteraceae
3	0.0003514	d_Bacteria,p_Proteobacteria,c_Alphaproteobacteria,o_Rhodobacterales,f_Rhodobacteraceae,uncl_f_Rhodobacteraceae
3	0.0002670	d_Bacteria,p_Proteobacteria,c_Alphaproteobacteria,o_Rhodobacterales,f_Rhodobacteraceae,uncl_f_Rhodobacteraceae
3	0.0002383	d_Bacteria,p_Proteobacteria,c_Alphaproteobacteria,o_Rhodobacterales,f_Rhodobacteraceae,uncl_f_Rhodobacteraceae
3	0.0002336	d_Bacteria,p_Proteobacteria,c_Alphaproteobacteria,o_Rhodobacterales,f_Rhodobacteraceae,uncl_f_Rhodobacteraceae
3	0.0002288	d_Bacteria,p_Proteobacteria,uncl_p_Proteobacteria,uncl_p_Proteobacteria,uncl_p_Proteobacteria,uncl_p_Proteobacteria
3	0.0002146	d_Bacteria,p_Proteobacteria,c_Alphaproteobacteria,o_Rhodobacterales,f_Rhodobacteraceae,uncl_f_Rhodobacteraceae
3	0.0001574	d_Bacteria,p_Bacteroidetes,c_Flavobacteriia,uncl_c_Flavobacteriia,uncl_c_Flavobacteriia,uncl_c_Flavobacteriia
3	0.0001509	d_Bacteria,p_Proteobacteria,c_Alphaproteobacteria,o_Rhodobacterales,f_Rhodobacteraceae,uncl_f_Rhodobacteraceae
3	0.0001477	d_Bacteria,p_Proteobacteria,c_Alphaproteobacteria,o_Rhodobacterales,f_Rhodobacteraceae,uncl_f_Rhodobacteraceae
3	0.0001473	d_Bacteria,p_Proteobacteria,c_Alphaproteobacteria,o_Rhodobacterales,f_Rhodobacteraceae,g_Profundibacterium
3	0.0001286	d_Bacteria,p_Proteobacteria,c_Alphaproteobacteria,o_Rhodobacterales,f_Rhodobacteraceae,g_Ruegeria
3	0.0001061	d_Bacteria,p_Proteobacteria,uncl_p_Proteobacteria,uncl_p_Proteobacteria,uncl_p_Proteobacteria,uncl_p_Proteobacteria
3	0.0000285	d_Bacteria,p_Proteobacteria,c_Alphaproteobacteria,o_Rhodobacterales,f_Rhodobacteraceae,uncl_f_Rhodobacteraceae
4	0.1928165	d_Bacteria,p_Proteobacteria,c_Gammaproteobacteria,o_Vibrionales,f_Vibrionaceae,g_Vibrio
4	0.0092669	d_Bacteria,p_Proteobacteria,c_Gammaproteobacteria,o_Alteromonadales,f_Pseudoalteromonadaceae,g_Pseudoalteromonas
4	0.0064387	d_Bacteria,p_Proteobacteria,c_Gammaproteobacteria,o_Alteromonadales,f_Colwelliaceae,g_Colwellia
4	0.0034041	d_Bacteria,p_Proteobacteria,c_Gammaproteobacteria,o_Alteromonadales,f_Alteromonadales_incertae_sedis,g_Psychrobium
4	0.0023363	d_Bacteria,p_Proteobacteria,c_Gammaproteobacteria,uncl_c_Gammaproteobacteria,uncl_c_Gammaproteobacteria,uncl_c_Gammaproteobacteria
4	0.0019401	d_Bacteria,p_Proteobacteria,c_Gammaproteobacteria,o_Vibrionales,f_Vibrionaceae,g_Aliivibrio
4	0.0018146	d_Bacteria,p_Proteobacteria,c_Alphaproteobacteria,o_Rhodobacterales,f_Rhodobacteraceae,uncl_f_Rhodobacteraceae
4	0.0013709	d_Bacteria,p_Proteobacteria,c_Gammaproteobacteria,o_Vibrionales,f_Vibrionaceae,uncl_f_Vibrionaceae
4	0.0013310	d_Bacteria,p_Proteobacteria,c_Gammaproteobacteria,o_Alteromonadales,f_Colwelliaceae,uncl_f_Colwelliaceae
4	0.0010668	d_Bacteria,p_Proteobacteria,c_Gammaproteobacteria,uncl_c_Gammaproteobacteria,uncl_c_Gammaproteobacteria,uncl_c_Gammaproteobacteria
4	0.0010188	d_Bacteria,p_Proteobacteria,c_Gammaproteobacteria,o_Vibrionales,uncl_o_Vibrionales,uncl_o_Vibrionales
4	0.0008171	d_Bacteria,p_Proteobacteria,c_Gammaproteobacteria,o_Alteromonadales,uncl_o_Alteromonadales,uncl_o_Alteromonadales
4	0.0006279	d_Bacteria,p_Proteobacteria,c_Alphaproteobacteria,uncl_c_Alphaproteobacteria,uncl_c_Alphaproteobacteria,uncl_c_Alphaproteobacteria
4	0.0005313	d_Bacteria,p_Proteobacteria,c_Gammaproteobacteria,o_Alteromonadales,f_Colwelliaceae,g_Colwellia
4	0.0005099	d_Bacteria,p_Proteobacteria,c_Gammaproteobacteria,o_Alteromonadales,f_Colwelliaceae,g_Colwellia
4	0.0004818	d_Bacteria,p_Proteobacteria,c_Gammaproteobacteria,o_Vibrionales,f_Vibrionaceae,g_Vibrio
4	0.0001578	d_Bacteria,p_Proteobacteria,c_Gammaproteobacteria,o_Alteromonadales,uncl_o_Alteromonadales,uncl_o_Alteromonadales
4	0.0001079	d_Bacteria,p_Proteobacteria,c_Alphaproteobacteria,o_Rhodobacterales,f_Rhodobacteraceae,uncl_f_Rhodobacteraceae
4	0.0001072	d_Bacteria,p_Proteobacteria,c_Gammaproteobacteria,o_Alteromonadales,uncl_o_Alteromonadales,uncl_o_Alteromonadales
4	0.0000983	d_Bacteria,p_Proteobacteria,c_Gammaproteobacteria,uncl_c_Gammaproteobacteria,uncl_c_Gammaproteobacteria,uncl_c_Gammaproteobacteria
4	0.0000546	d_Bacteria,p_Proteobacteria,c_Gammaproteobacteria,o_Alteromonadales,uncl_o_Alteromonadales,uncl_o_Alteromonadales

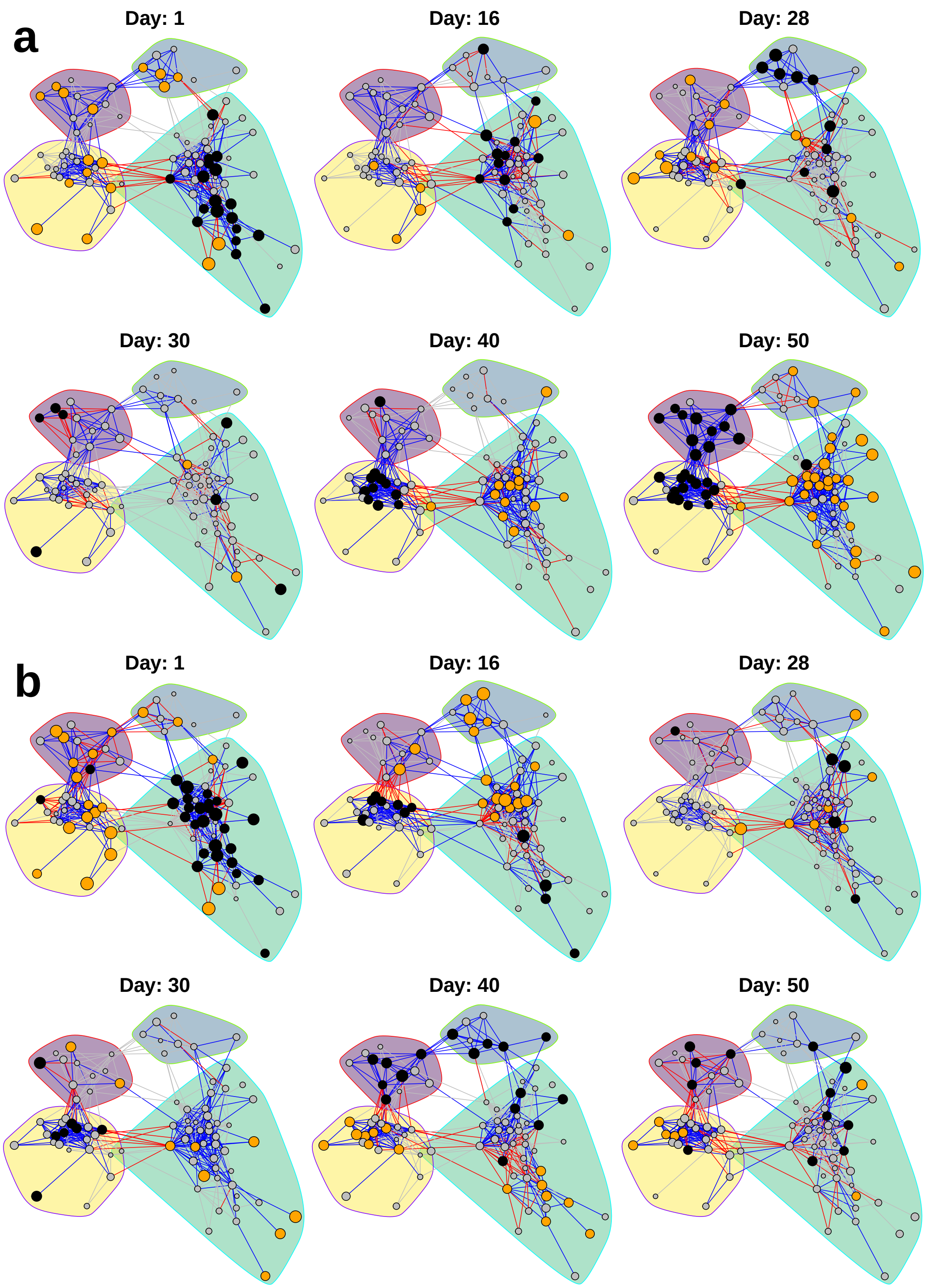

Figure S1. Dynamic visualisation of the network in figure 2, for **a)** the RK selection group and **b)** the KR selection group for low (L) nutrient supply. Nodes are coloured according to the corresponding OTUs' abundance compared to its overall mean for all sampling days, represented by its z-score. Orange, grey and black nodes mean higher, about the same or lower abundance than its mean, respectively. The edges are coloured by the product of the nodes' z-scores. This means that blue and red edges contribute to positive and negative association across the time series, respectively. The grey edges indicate that no major contribution to neither positive nor negative association is made. As we want to emphasize the orange and black nodes, the nodes with higher absolute z-scores are larger.

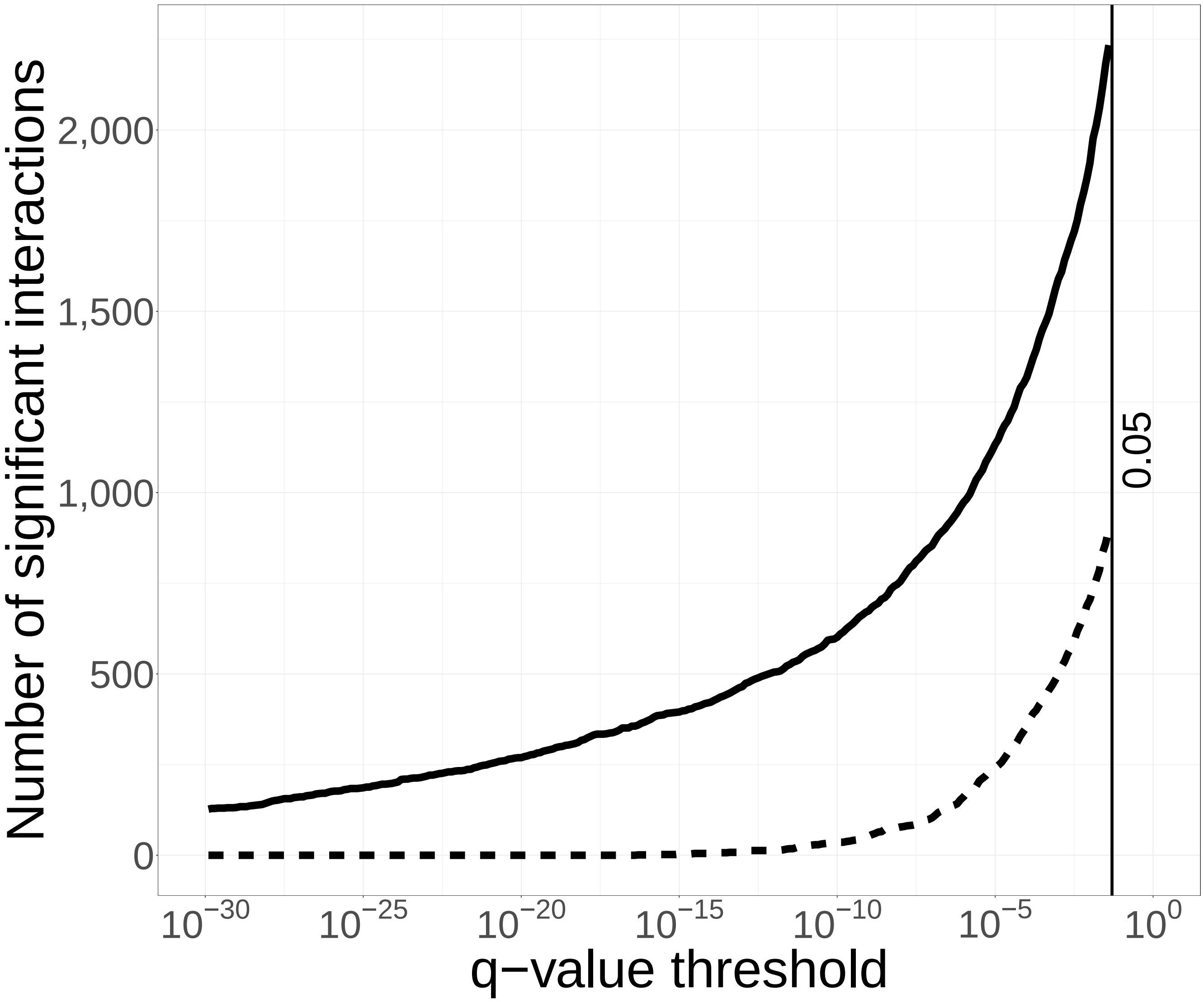

Figure S2. The cumulative number of significant interactions as a function of the critical q-value threshold considered. The solid line signifies positive interactions detected, while the dashed line represents negative interactions.

S2 Robustness to random noise

This subsection shows the results when the default low level of random noise with magnitude factor $s = 1$ is replaced with medium level of random noise with magnitude factor $s = 10$ and all other parameters kept constant. The cumulative of number of interactions versus q-value threshold is shown in Fig. S2. The network of the 500 most significant associations is shown in Fig. S3 with the corresponding phylogentic tree shown in Fig. S4. The PCoA ordinations of the time trajectories are shown in Fig. S5. Finally, the dynamic visualisation of the network in Fig. S3 is shown in Fig. S6.

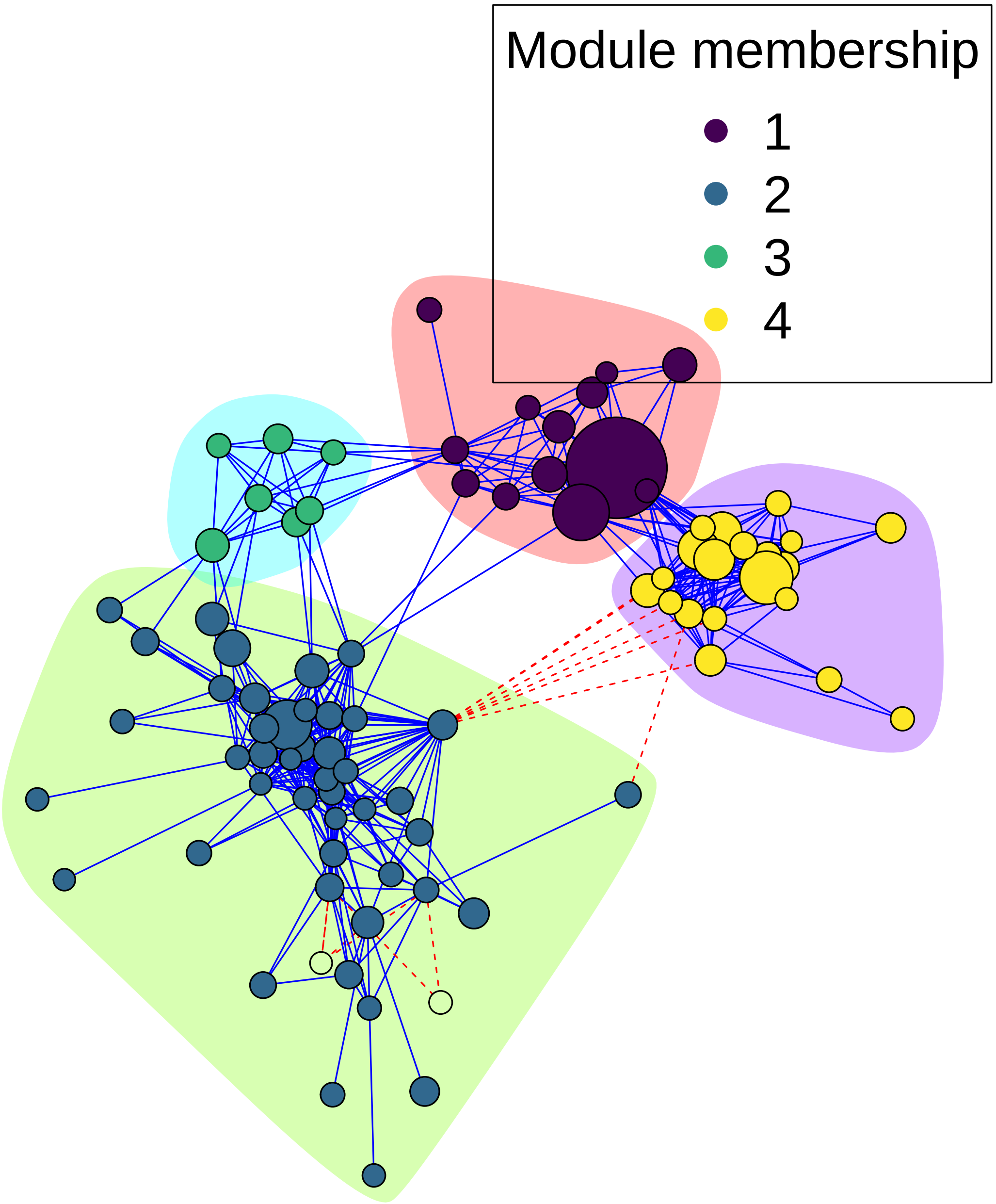

Figure S3. Module labelled network of the 500 most significant interactions in the r/K-selection-switch dataset. Each of the 87 nodes is an OTU, while each edge is a significant association between the OTUs. Blue solid edges indicate positive interactions, whereas red dashed edges indicate negative interactions. The nodes are sized according to their overall mean abundance.

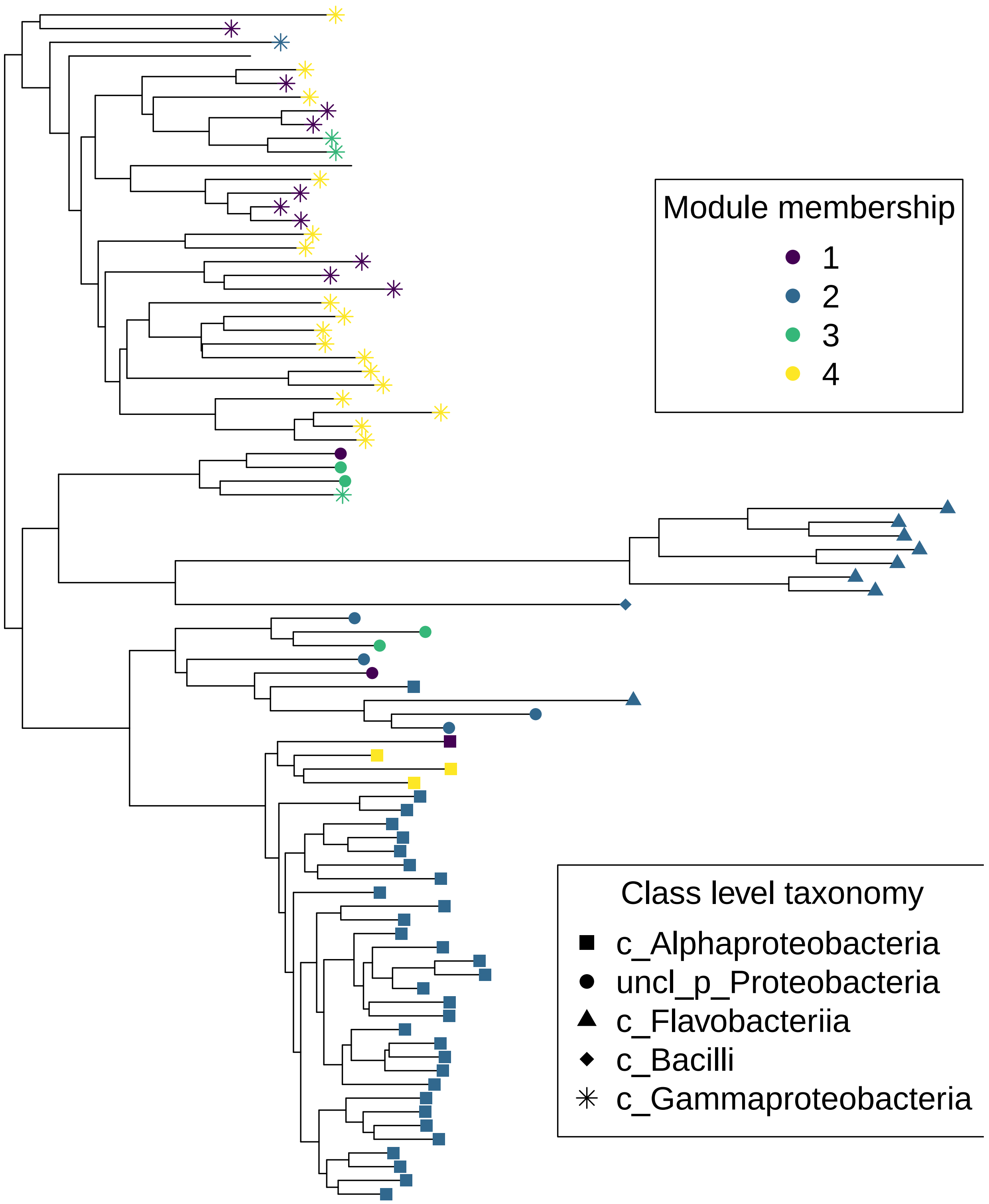

Figure S4. The phylogentic tree of the 87 OTUs from Fig. S3 together with the class level taxonomical assignment. Point colour indicates module membership, whereas the shape indicates class level taxonomical assignment. Notice that there are some inconsistencies between the phylogenetic tree and the assigned taxonomy.

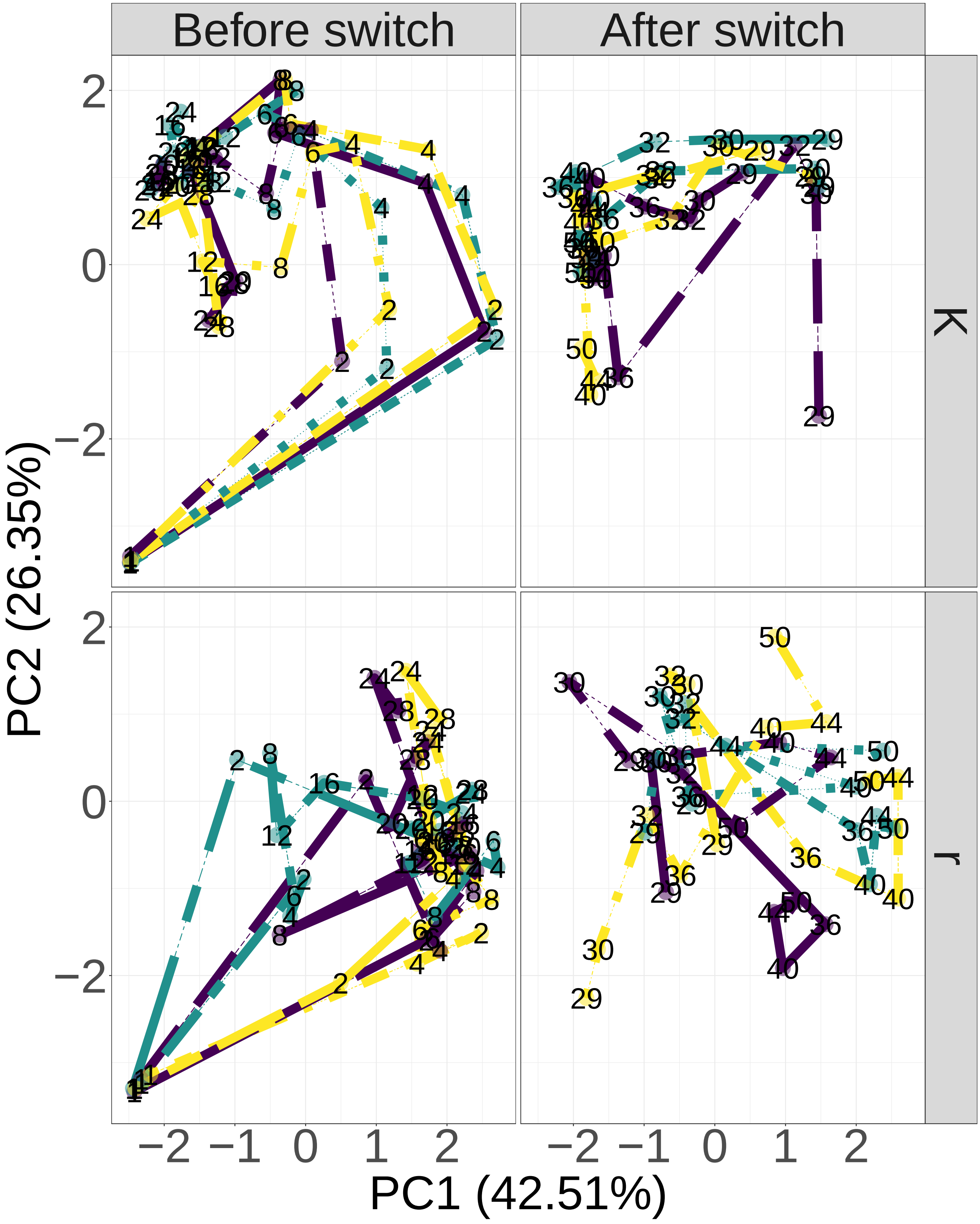

Figure S5. PCoA ordination of Bray-Curtis distances between samples showing the time trajectories for each mesocosm. The vertical facets show the selection regime at sampling, *K* or *r*. Solid and dotted lines indicate high (H) and low (L) nutrient supply, respectively. The labels indicate the day of sampling, whereas the line colours are purely to visually distinguish the replicate time series.

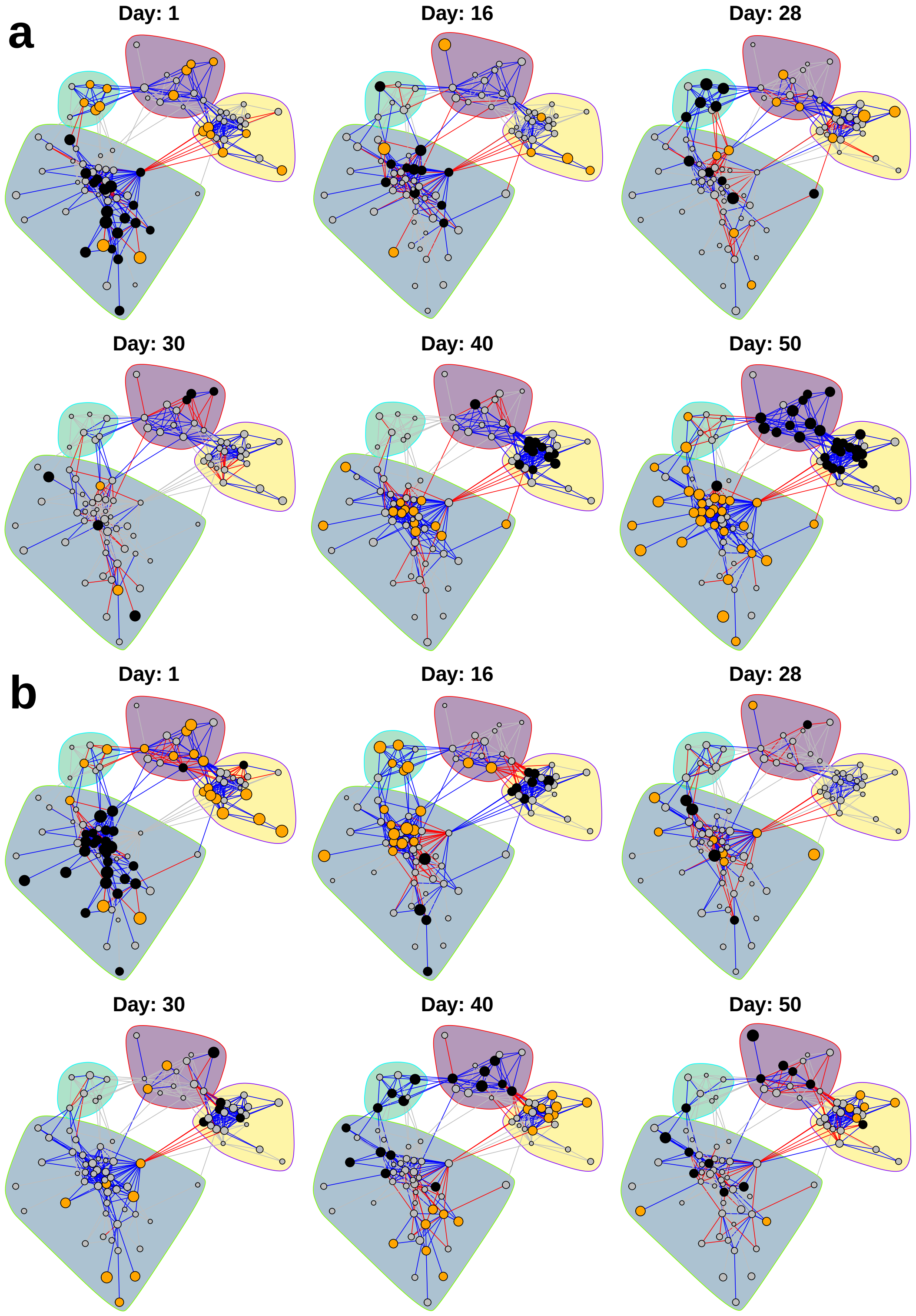

Figure S6. Dynamic visualisation of the network in figure S3, for **a)** the RK selection group and **b)** the KR selection group for high (H) nutrient supply. Nodes are coloured according to the corresponding OTUs' abundance compared to its overall mean for all sampling days, represented by its z-score. Orange, grey and black nodes mean higher, about the same or lower abundance than its mean, respectively. The edges are coloured by the product of the nodes' z-scores. This means that blue and red edges contribute to positive and negative association across the time series, respectively. The grey edges indicate that no major contribution to neither positive nor negative association is made. As we want to emphasize the orange and black nodes, the nodes with higher absolute z-scores are larger.

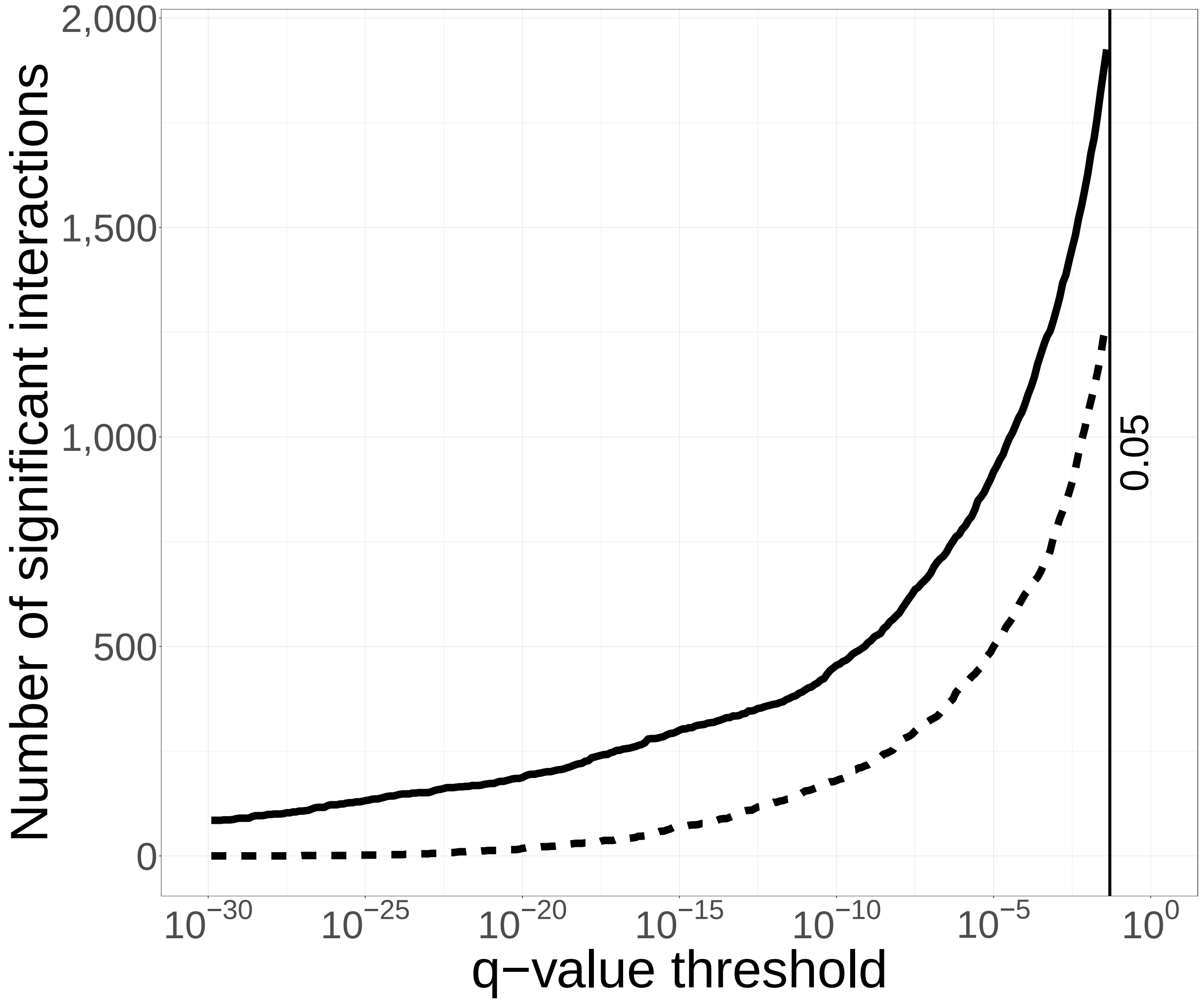

Figure S7. The cumulative number of significant interactions as a function of the critical q-value threshold considered. The solid line signifies positive interactions detected, while the dashed line represents negative interactions.

S3 Robustness to change of abundance type

This subsection shows the results when the absolute abundances used in the main article are replaced by relative abundances and all other parameters kept constant. The cumulative of number of interactions versus q-value threshold is shown in Fig. S7. The network of the 500 most significant associations is shown in Fig. S8 with the corresponding phylogentic tree shown in Fig. S9. The PCoA ordinations of the time trajectories are shown in Fig. S10. Finally, the dynamic visualisation of the network in Fig. S8 is shown in Fig. S11.

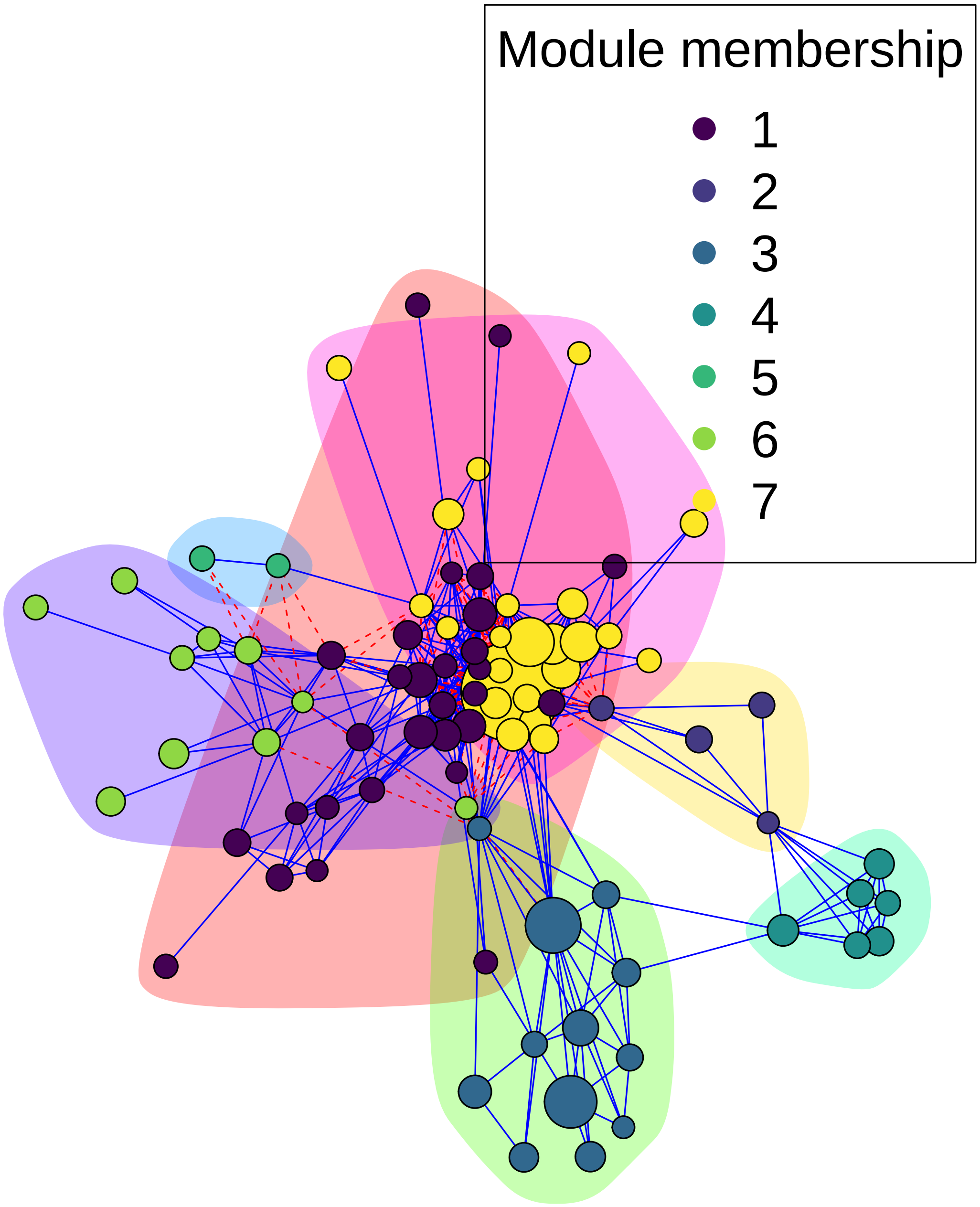

Figure S8. Module labelled network of the 500 most significant interactions in the *r*/*K*-selection-switch dataset. Each of the 86 nodes is an OTU, while each edge is a significant association between the OTUs. Blue solid edges indicate positive interactions, whereas red dashed edges indicate negative interactions. The nodes are sized according to their overall mean abundance.

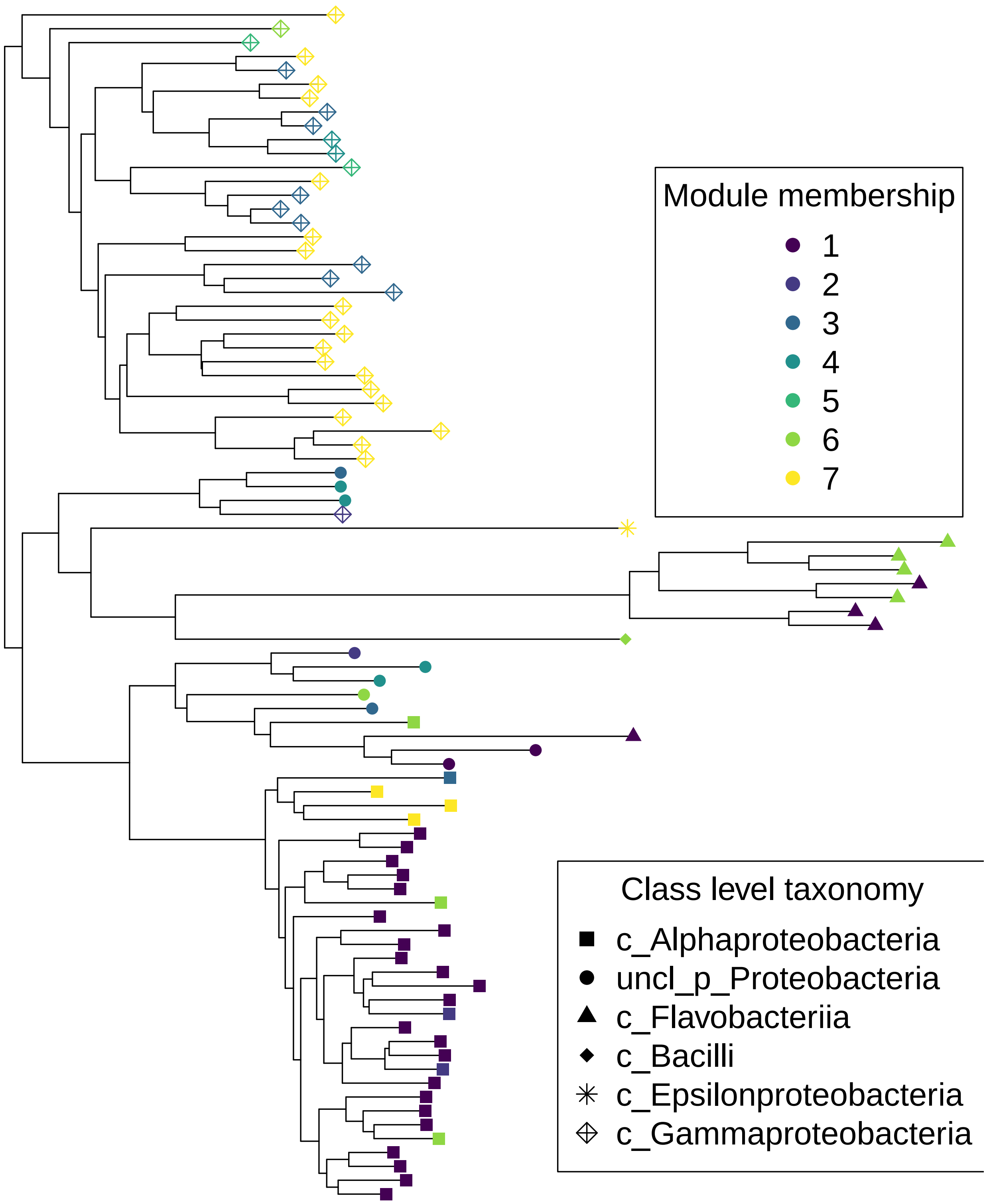

Figure S9. The phylogentic tree of the 86 OTUs from Fig. S8 together with the class level taxonomical assignment. Point colour indicates module membership, whereas the shape indicates class level taxonomical assignment. Notice that there are some inconsistencies between the phylogenetic tree and the assigned taxonomy.

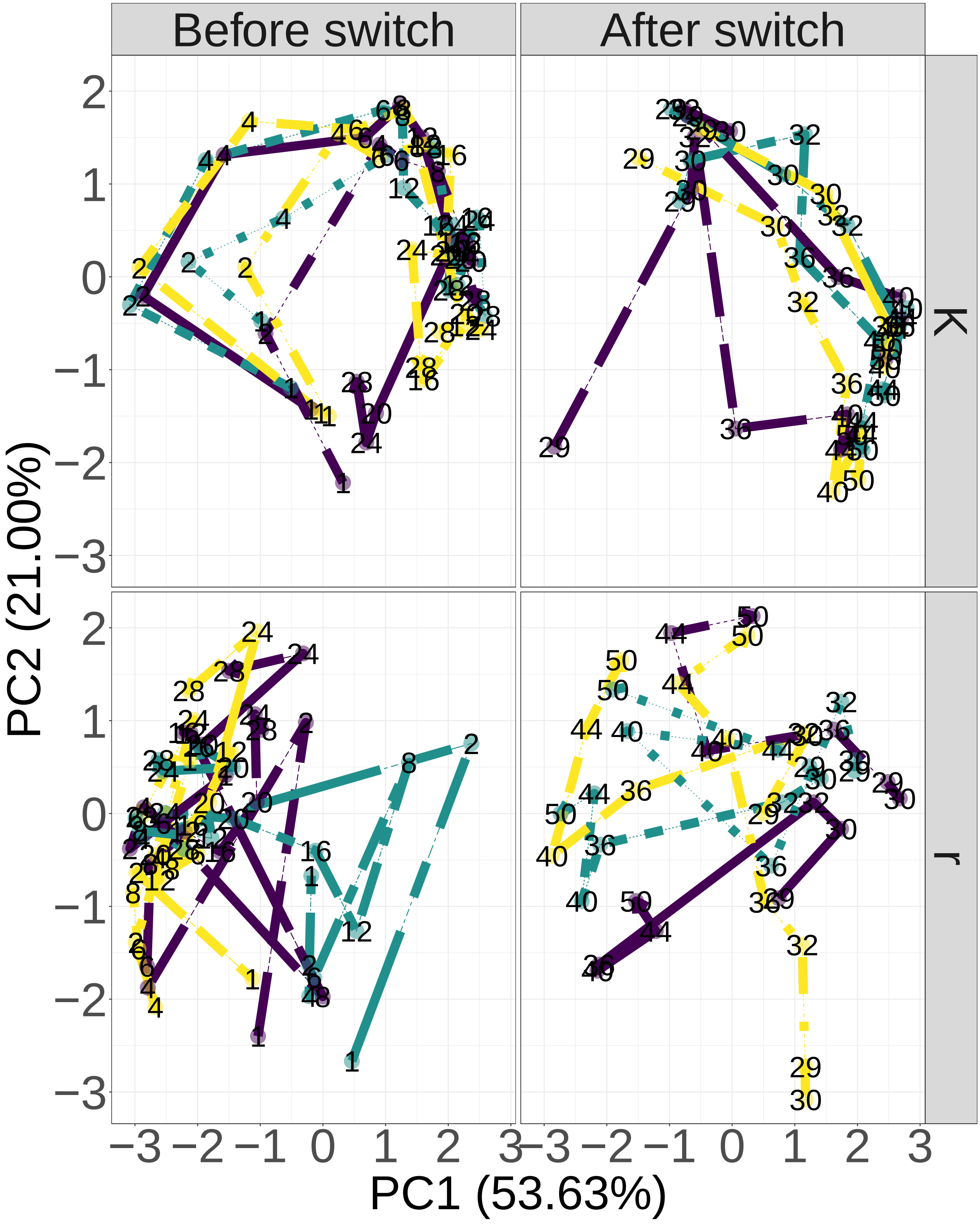

Figure S10. PCoA ordination of Bray-Curtis distances between samples showing the time trajectories for each mesocosm. The vertical facets show the selection regime at sampling, K or r. Solid and dotted lines indicate high (H) and low (L) nutrient supply, respectively. The labels indicate the day of sampling, whereas the line colours are purely to visually distinguish the replicate time series.

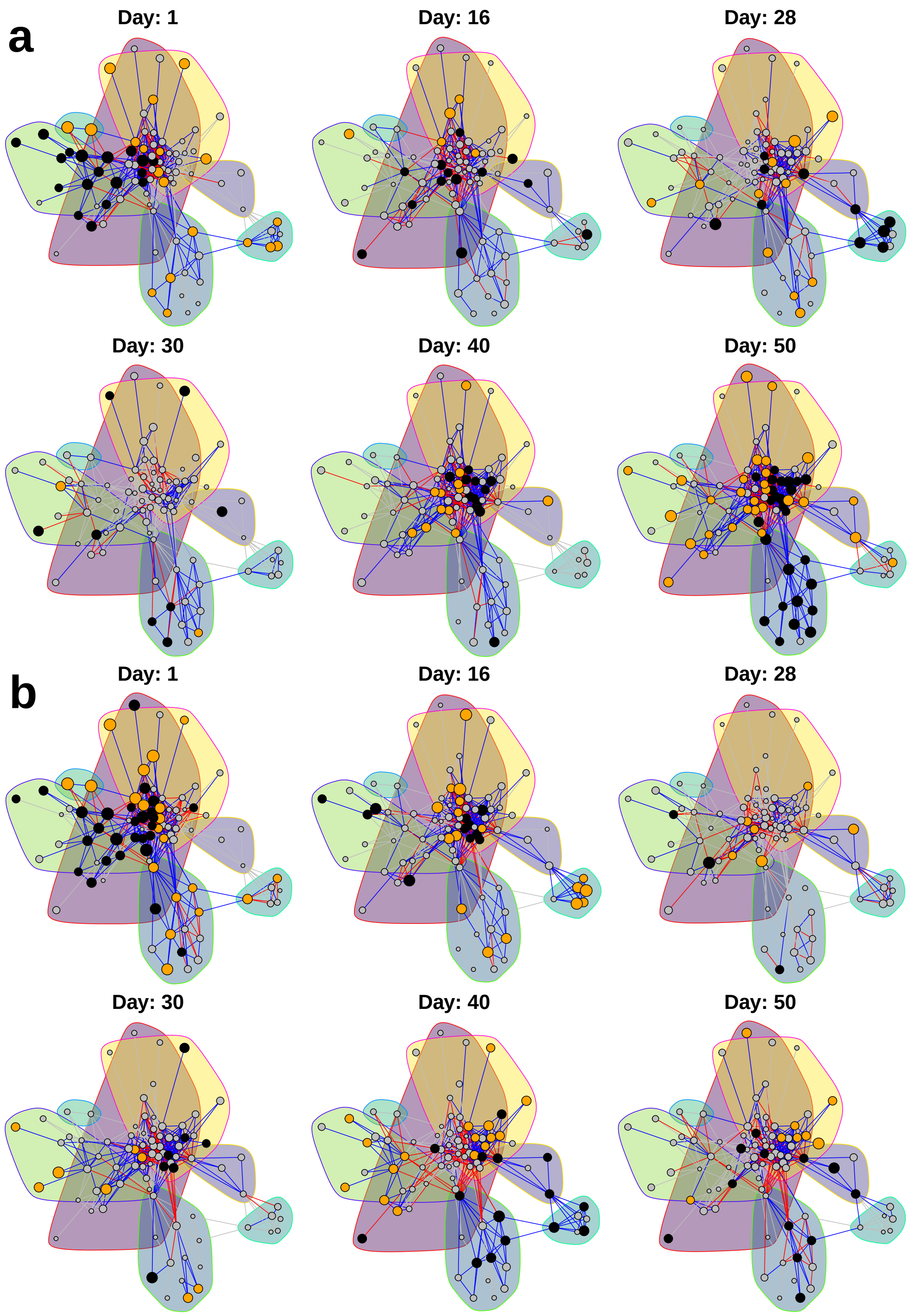

Figure S11. Dynamic visualisation of the network in figure S8, for **a)** the RK selection group and **b)** the KR selection group for high (H) nutrient supply. Nodes are coloured according to the corresponding OTUs' abundance compared to its overall mean for all sampling days, represented by its z-score. Orange, grey and black nodes mean higher, about the same or lower abundance than its mean, respectively. The edges are coloured by the product of the nodes' z-scores. This means that blue and red edges contribute to positive and negative association across the time series, respectively. The grey edges indicate that no major contribution to neither positive nor negative association is made. As we want to emphasize the orange and black nodes, the nodes with higher absolute z-scores are larger.

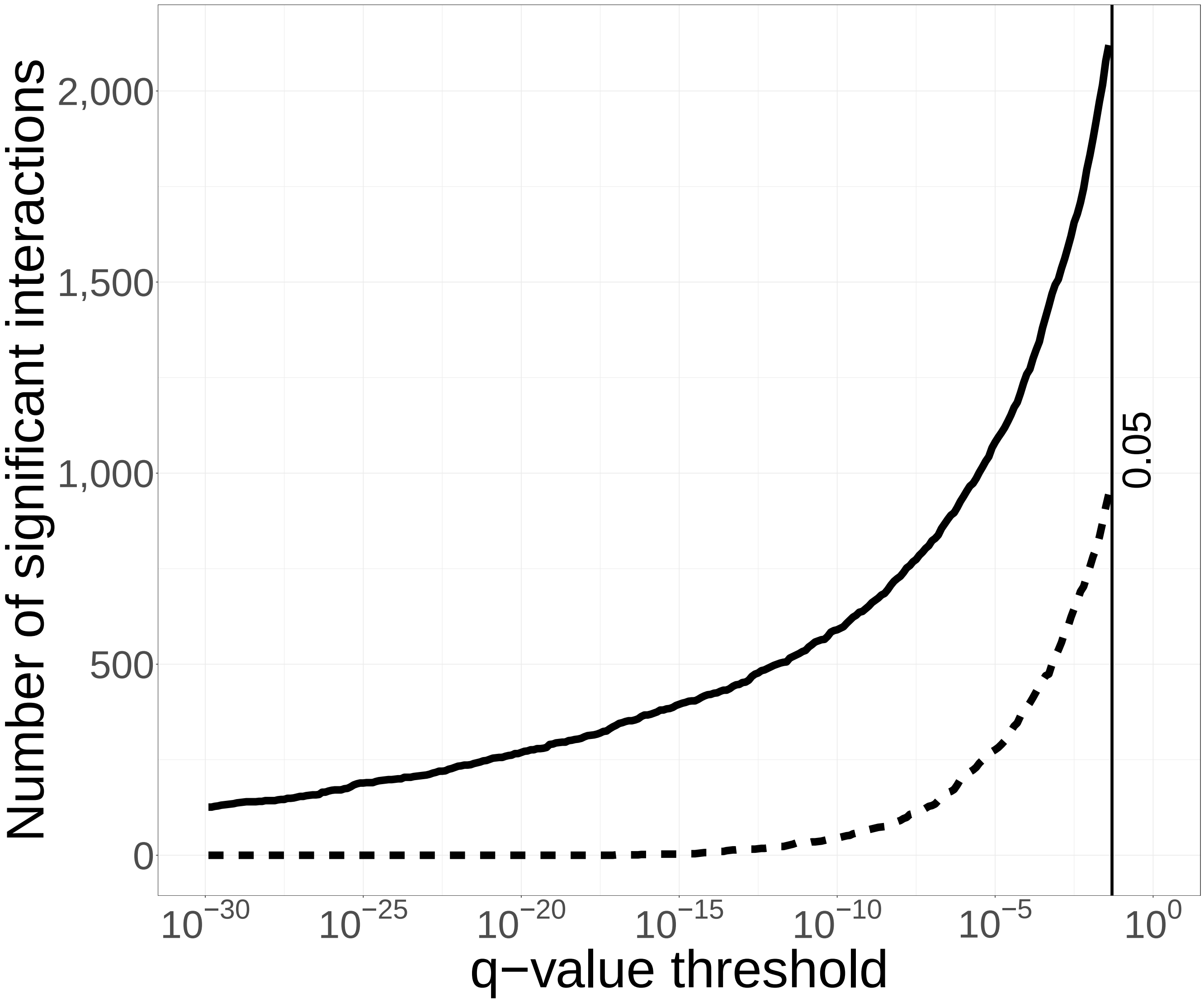

Figure S12. The cumulative number of significant interactions as a function of the critical q-value threshold considered. The solid line signifies positive interactions detected, while the dashed line represents negative interactions.

S4 Robustness to OTU filtering cutoff

This subsection shows the results when the OTU filtering cut-off is increased from low level at $5 \cdot 10^{-4}$ maximum relative abundance to $1 \cdot 10^{-3}$ maximum relative abundance. All other parameters in the main article are kept constant. The cumulative of number of interactions versus q-value threshold is shown in Fig. S12. The network of the 500 most significant associations is shown in Fig. S13 with the corresponding phylogentic tree shown in Fig. S14. The PCoA ordinations of the time trajectories are shown in Fig. S15. Finally, the dynamic visualisation of the network in Fig. S13 is shown in Fig. S16.

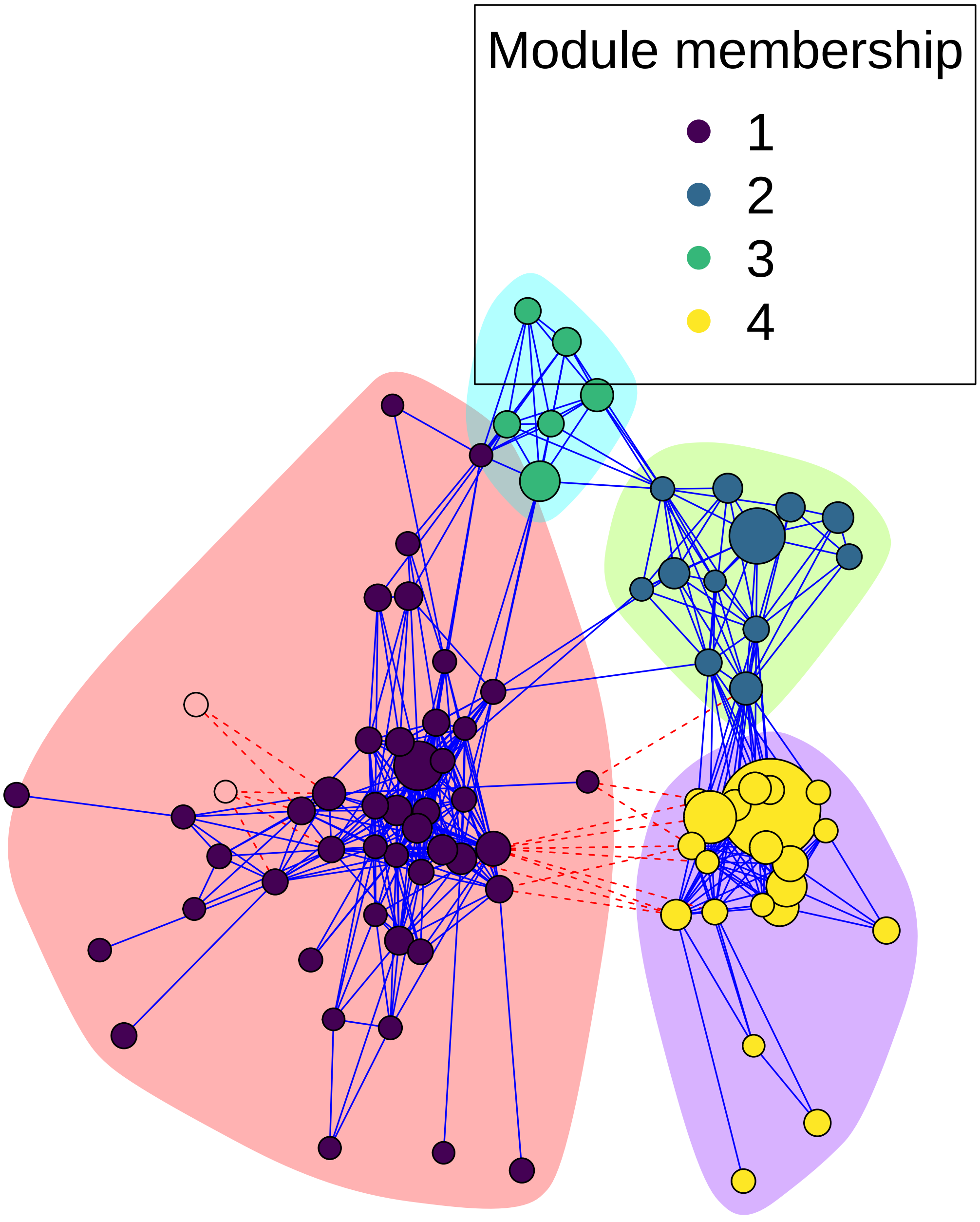

Figure S13. Module labelled network of the 500 most significant interactions in the r/K-selection-switch dataset. Each of the 86 nodes is an OTU, while each edge is a significant association between the OTUs. Blue solid edges indicate positive interactions, whereas red dashed edges indicate negative interactions. The nodes are sized according to their overall mean abundance.

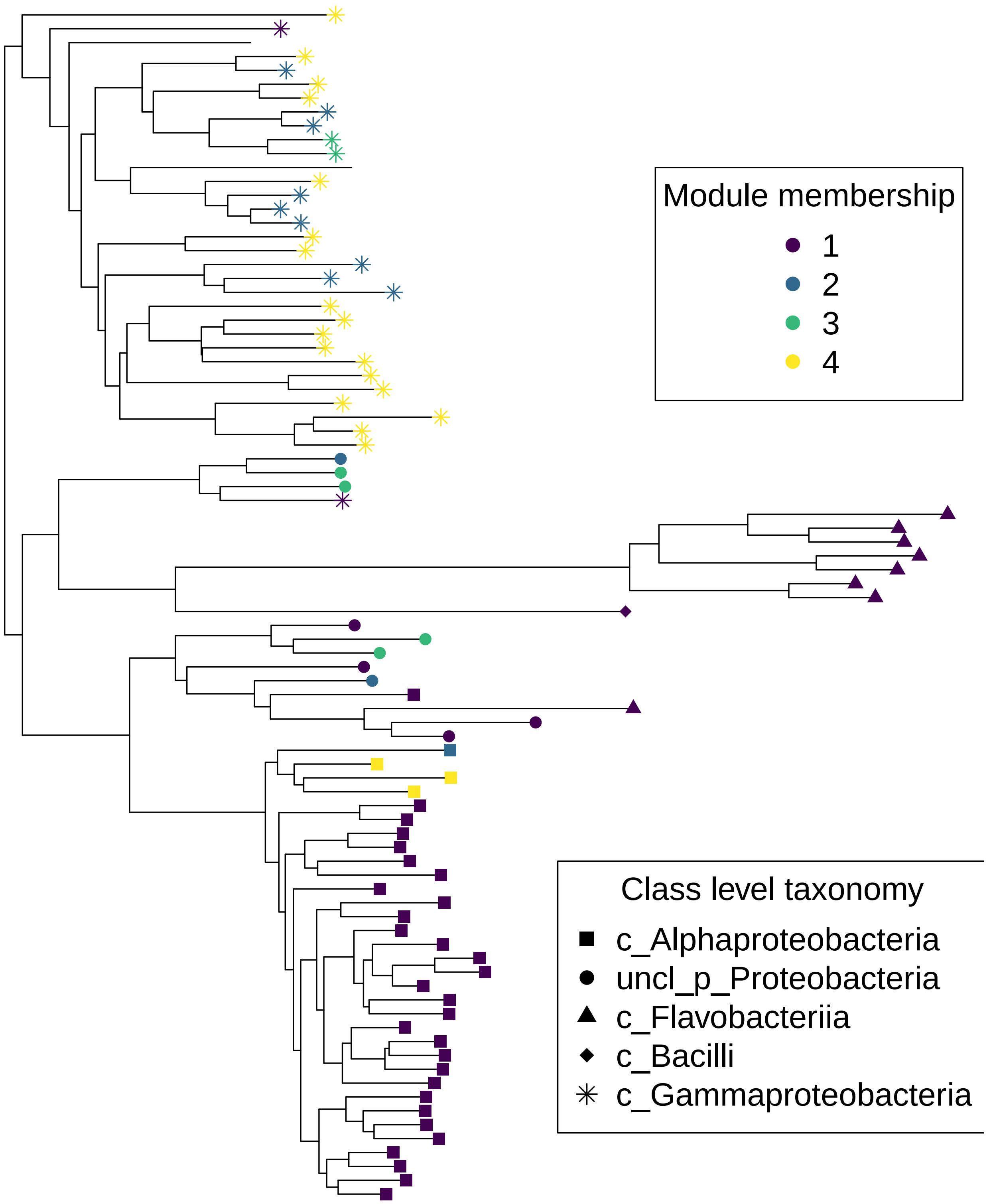

Figure S14. The phylogentic tree of the 86 OTUs from Fig. S13 together with the class level taxonomical assignment. Point colour indicates module membership, whereas the shape indicates class level taxonomical assignment. Notice that there are some inconsistencies between the phylogenetic tree and the assigned taxonomy.

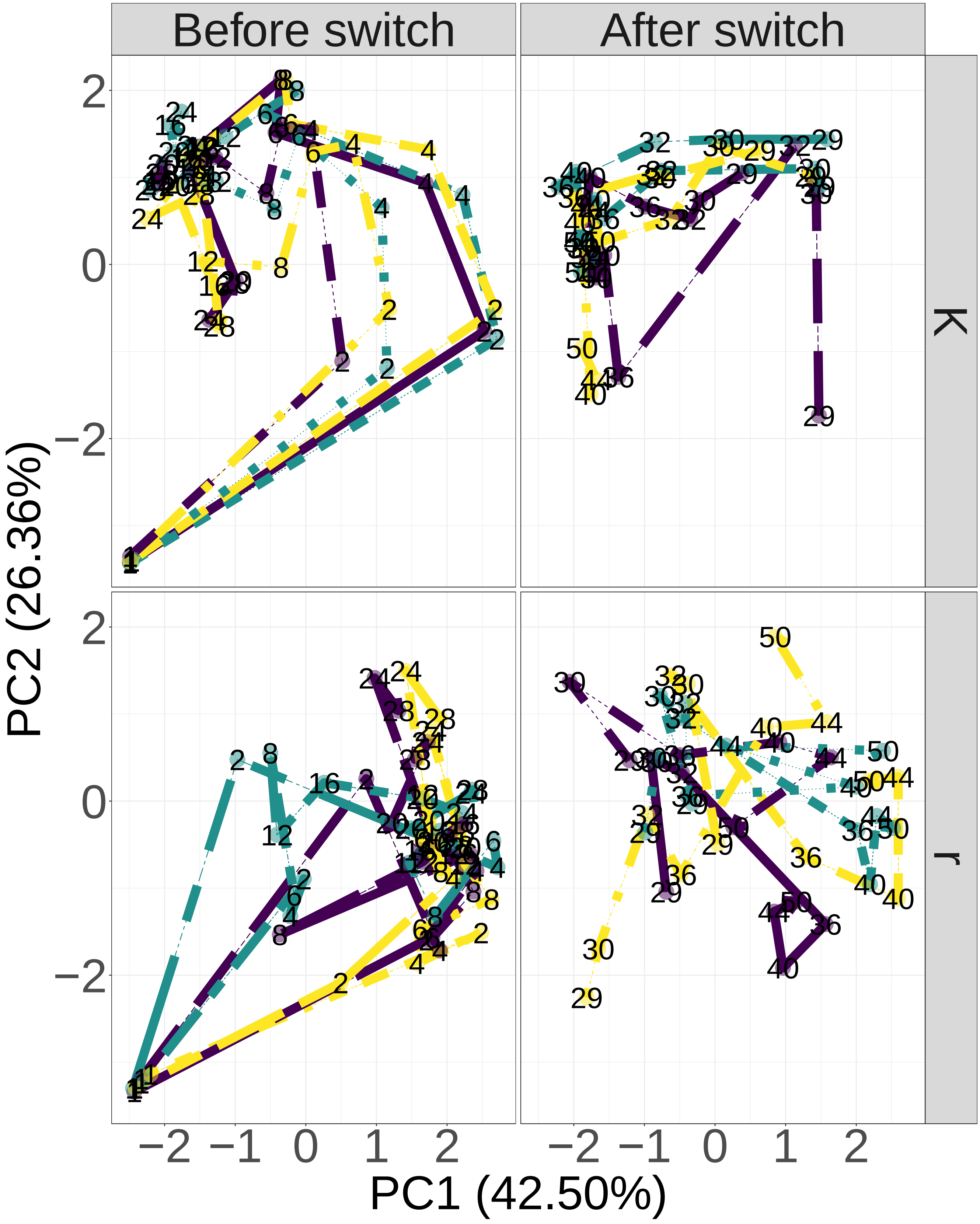

Figure S15. PCoA ordination of Bray-Curtis distances between samples showing the time trajectories for each mesocosm. The vertical facets show the selection regime at sampling, *K* or *r*. Solid and dotted lines indicate high (H) and low (L) nutrient supply, respectively. The labels indicate the day of sampling, whereas the line colours are purely to visually distinguish the replicate time series.

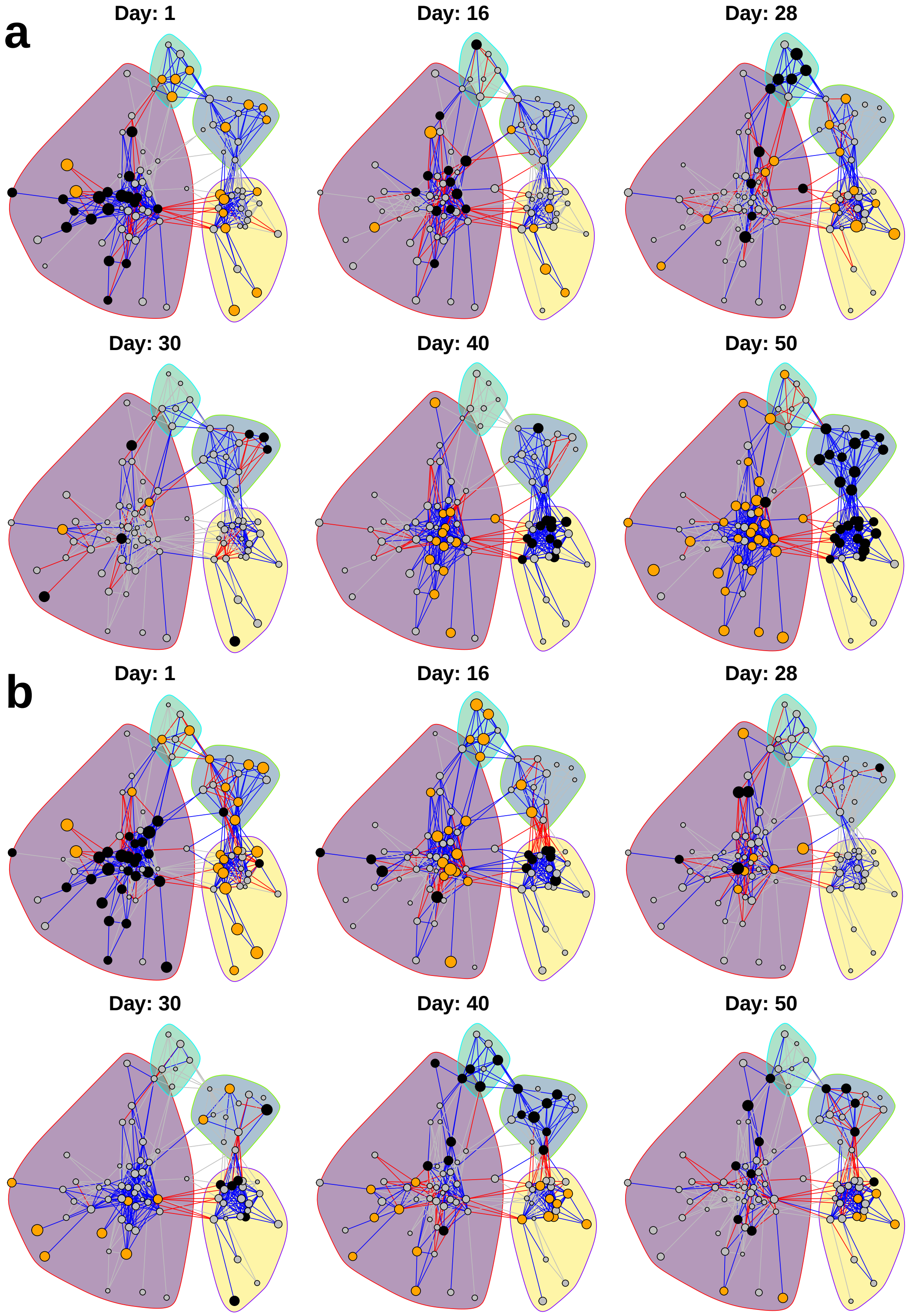

Figure S16. Dynamic visualisation of the network in figure S13, for **a)** the RK selection group and **b)** the KR selection group for high (H) nutrient supply. Nodes are coloured according to the corresponding OTUs' abundance compared to its overall mean for all sampling days, represented by its z-score. Orange, grey and black nodes mean higher, about the same or lower abundance than its mean, respectively. The edges are coloured by the product of the nodes' z-scores. This means that blue and red edges contribute to positive and negative association across the time series, respectively. The grey edges indicate that no major contribution to neither positive nor negative association is made. As we want to emphasize the orange and black nodes, the nodes with higher absolute z-scores are larger.

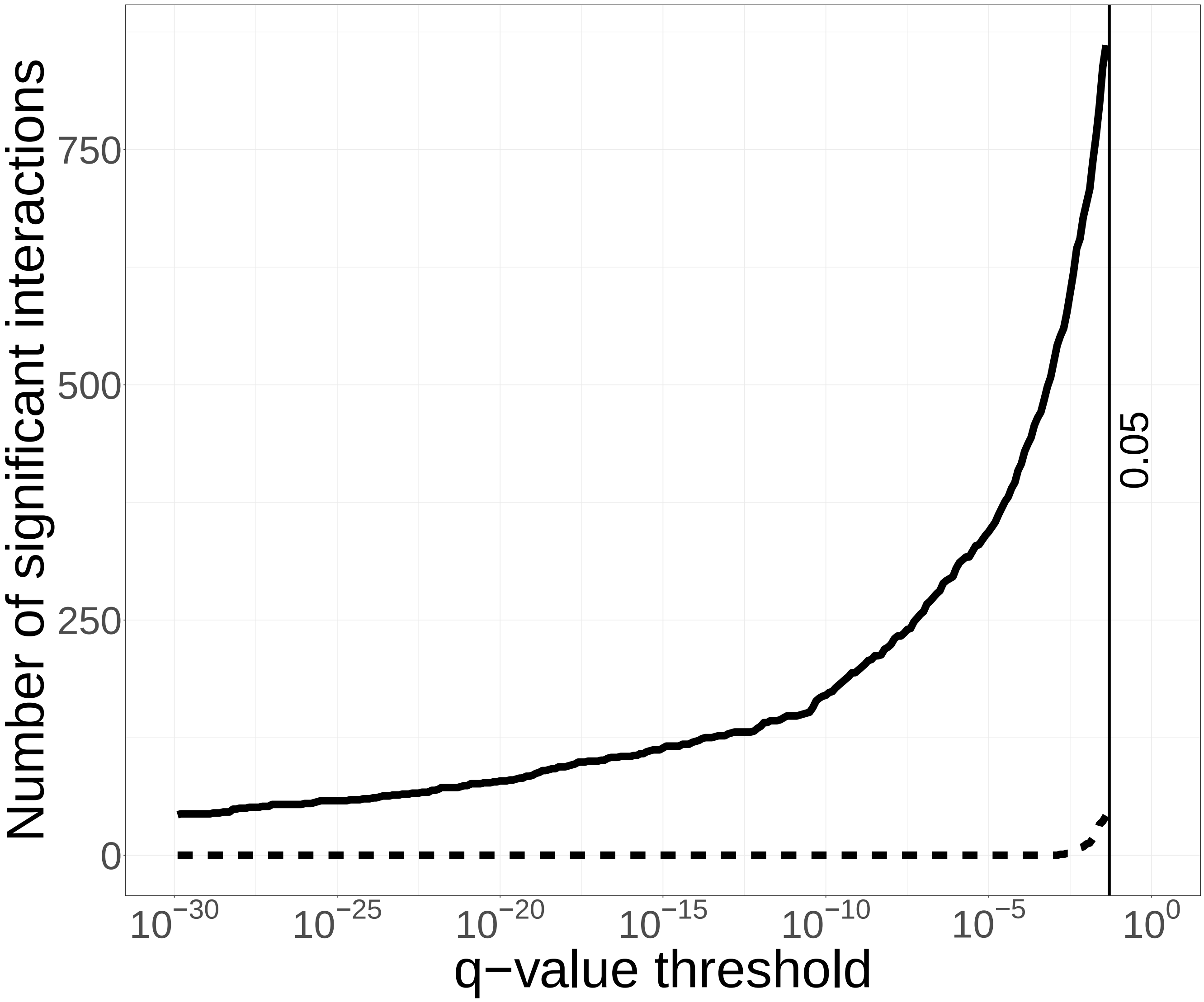

Figure S17. The cumulative number of significant interactions as a function of the critical q-value threshold considered. The solid line signifies positive interactions detected, while the dashed line represents negative interactions.

S5 Effect of similarity measure

This subsection shows the results when the rank-based Spearman correlation is replaced by the linear Pearson correlation. The cumulative of number of interactions versus q-value threshold is shown in Fig. S17. The network of the 500 most significant associations is shown in Fig. S18 with the corresponding phylogentic tree shown in Fig. S19. The PCoA ordinations of the time trajectories are independent of the similarity measures for inferring interactions and is therefore omitted. Finally, the dynamic visualisation of the network in Fig. S18 is shown in Fig. S20.

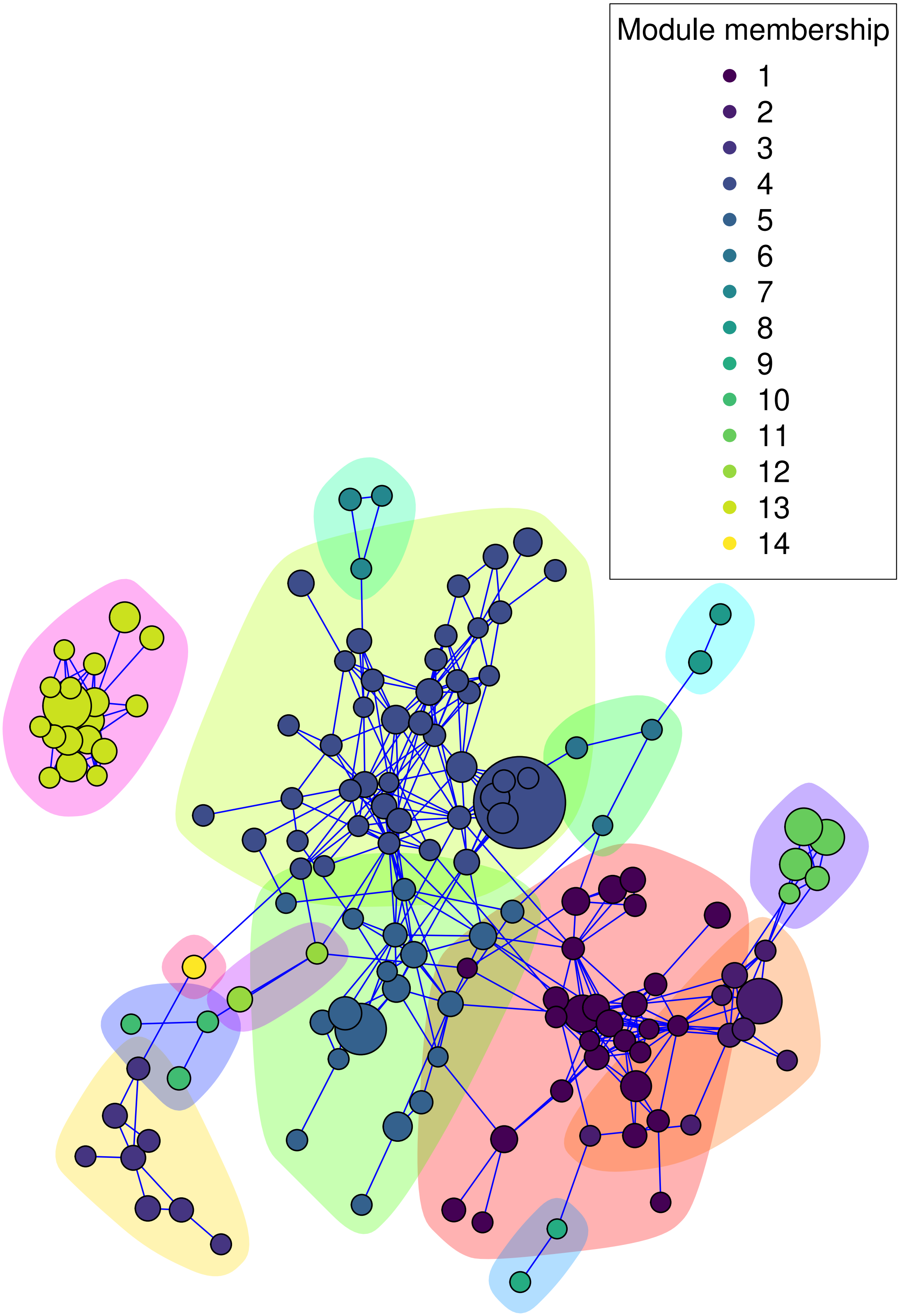

Figure S18. Module labelled network of the 500 most significant interactions in the r/K-selection-switch dataset. Each of the 147 nodes is an OTU, while each edge is a significant association between the OTUs. Blue solid edges indicate positive interactions, whereas red dashed edges indicate negative interactions. The nodes are sized according to their overall mean abundance.

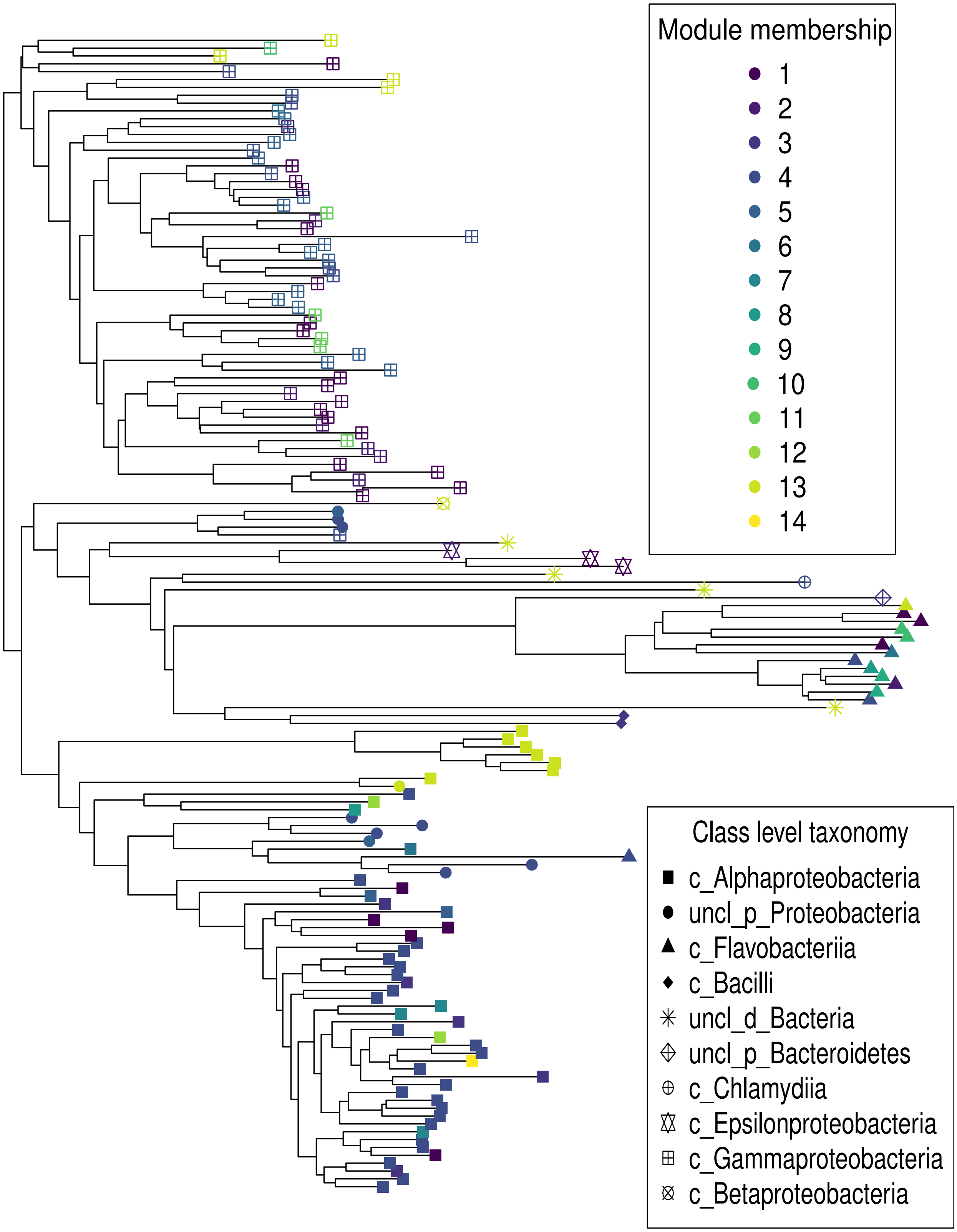

Figure S19. The phylogentic tree of the 147 OTUs from Fig. S18 together with the class level taxonomical assignment. Point colour indicates module membership, whereas the shape indicates class level taxonomical assignment. Notice that there are some inconsistencies between the phylogenetic tree and the assigned taxonomy.

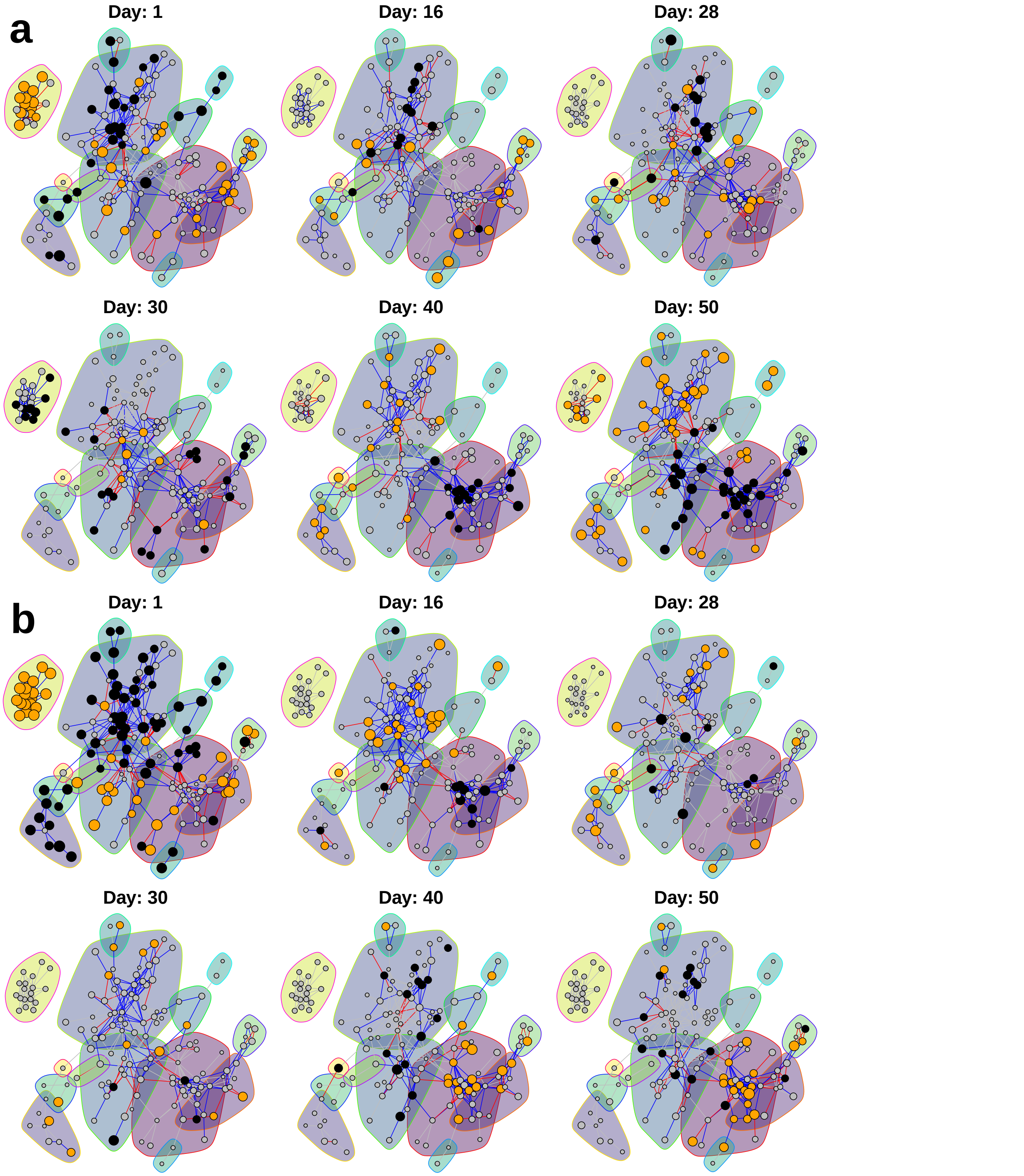

Figure S20. Dynamic visualisation of the network in figure S18, for **a)** the RK selection group and **b)** the KR selection group for high (H) nutrient supply. Nodes are coloured according to the corresponding OTUs' abundance compared to its overall mean for all sampling days, represented by its z-score. Orange, grey and black nodes mean higher, about the same or lower abundance than its mean, respectively. The edges are coloured by the product of the nodes' z-scores. This means that blue and red edges contribute to positive and negative association across the time series, respectively. The grey edges indicate that no major contribution to neither positive nor negative association is made. As we want to emphasize the orange and black nodes, the nodes with higher absolute z-scores are larger.
